# Silicon-based mid infrared on-chip gas sensor using Fano resonance of coupled plasmonic microcavities

**DOI:** 10.1038/s41598-023-38926-9

**Published:** 2023-07-29

**Authors:** Sherif M. Sherif, Mohamed A. Swillam

**Affiliations:** Department of Physics, School of Science and Engineering, The American University in Cairo, Cairo, 11835 Egypt

**Keywords:** Optical sensors, Microresonators, Mid-infrared photonics, Nanophotonics and plasmonics

## Abstract

Sensing in the mid infrared spectral range is highly desirable for the detection and monitoring of different gases. We hereby propose a CMOS compatible silicon-based sensor that operates at (3.5–10 μm) within the mid infrared range. The silicon material is doped to the level that shifts its plasmonic resonance to 3 μm wavelength. The sensor device comprises an in-line rectangular microcavity and a stub microcavity resonator. The resonance frequencies/wavelengths of the two resonators were studied with different design dimensions. When the two resonators are designed to resonate at close frequencies, the interesting Fano resonance with its distinct and sharp line shape is excited due to the interference between the two resonance profiles. Fano resonance is useful for highly sensitive measurements due to its abrupt intensity changing profile. The sensor is studied and analyzed using Finite Difference Element and 2D Finite Difference Time Domain methods. The sensor's performance is characterized by its high sensitivity of 6000 nm/RIU, FOM of 353, and limited insertion loss of 0.45 dB around 6.5 μm operation wavelength. Furthermore, we develop the sensor for simultaneously detecting formaldehyde CH_2_O and nitrous oxide N_2_O gases from their strong absorption bands at 3.6 μm and 4.46 μm wavelengths, respectively.

## Introduction

Mid infrared sensing is of special importance due to its applications in different domains such as telecommunication, defense, environmental and industrial monitoring, as many gases have their absorption fingerprints in the mid infrared range^1,2^. Optical sensors are being developed based on two main platforms: the conventional silicon Si photonic and plasmonic platforms^3^. While Si structures have the advantages of being CMOS compatible and having low waveguiding losses, plasmonic structures can have much smaller dimensions. Moreover, plasmonic structures possess the interesting properties of enhancing and confining the electromagnetic fields in small regions^4,5^ such as in metal–insulator-metal waveguides, plasmonic slots, and cavities. The issue with the commonly used noble metals Au and Ag is that they have a fixed density of free electrons resulting in a fixed plasmonic resonance frequency, in addition to being CMOS incompatible. On the contrary, doped semiconductors^6,7^ have the advantages of CMOS compatibility, and tunability of plasmonic resonance frequency with doping concentration^8^.

Sensors working principles are based on strong optical resonating and interference effects which are achieved in different configurations such as racetrack resonators^9^, and Mach Zender interferometers^10^. Other technologies include toroidal^11^, surface lattice resonance^12^, and bound state in the continuum sensors^13^ were also investigated. However, we would like to study the effect of coupled resonators in mid infrared sensing which can potentially improve sensors performance. Coupling two or more resonators can lead to peculiar properties and spectral line-shapes with special profiles such as the Fano resonance^14–16^, the Electromagnetically Induced Transparency, and the Borrmann effect^17^.

In general, Fano resonance is a phenomenon that occurs in integrated photonics where light waves interact with matter in a way that produces sharp dips or peaks in the transmission spectrum. The Fano resonance was first described by Italian physicist Ugo Fano in 1961^15^, and it has since been observed in various systems, including quantum dots, plasmonic nanoparticles, and photonic crystals.

The Fano resonance arises from the interference between two optical pathways. One pathway involves a direct transmission of light through the material, while the other involves scattering of light by a discrete resonant structure within the material. The interference between these two pathways can produce a constructive or destructive interference effect, leading to either a sharp peak or a dip in the transmission spectrum. This effect is highly sensitive to the properties of the resonant structure and can be used for a variety of sensing and signal processing applications.

The Fano resonance has been demonstrated in various integrated photonic structures, including waveguides, cavities, and resonators. These structures can be designed to have specific resonant frequencies, and the Fano resonance effect can be used to enhance or suppress the transmission of light at these frequencies. This has led to a wide range of potential applications, such as optical filters, sensors, and modulators, and it is an active area of research in the field of integrated photonics.

Fano resonance is promising in sensing applications since it possesses a distinct sharp line-shape. The excitation of Fano resonance is described by the interference of wide and narrow spectral lines or resonances^17^, which results in a redistribution of the electromagnetic fields in the microcavities.

Different materials and designs were investigated to develop state of the art Fano resonance-based sensors operating in the mid infrared range including graphene and Au nano-antenna arrays^18^, and nanodisks^19^, 1D photonic crystal structure composed of Al, Au, Ag, and Pt^20^, Ag nanorods^21^, Si Lucky knot structure^22^, and the performance of these sensors is compared to our Fano resonance based sensor in Table 1.

**Table 1:** Comparison between Fano resonance-based sensors in the mid infrared range.

Year [Ref]	Materials	Unit structure	l (mm)	Sensitivity (nm/RIU)	FOM (RIU)^-1^	Insertion loss (dB)
2019 ^15^	Au, and Graphene	Nano-antenna array	6.5	2300	28.75	4.5
2019 ^19^	Al, Au, Ag, and Pt	1D Photonic crystal	7	5018	1477.5	NA
2020 ^20^	Ag	Stub, and nanorods	3	5140	NA	0.96
2020 ^18^	Au, and Graphene	nanodisks	10	7930	158.7	2.2
2021 ^21^	Si	Lucky knot	7	986	32.7	NA
This work	Doped Si	Stub	6.5	6000	353	0.45

In this work, we introduce a Fano resonance based mid infrared sensor which achieves 6000 nm/RIU sensitivity at the 6.5 μm wavelength, and an insertion loss of 0.45 dB. We begin our investigation by analyzing the response of an in-line rectangular cavity resonator^23^ and its resonance profiles. Then we study the spectral response and the resonance orders of the stub cavity resonator. Then, we integrate the in-line and stub rectangular cavity resonators in the same device and optimize them to resonate at close frequencies and study the response of these coupled resonators. Finally, we develop the sensor for the detection of two gases on the same chip by exciting two Fano resonances that correspond to the CH_2_O and N_2_O gases strong absorption bands in the mid infrared at 3.6 μm and 4.46 μm^24,25^.

## Device structure

### Doped silicon model

To experience plasmonic effects in the mid infrared range, we use Si doped with phosphorus. The n-doped Si model is based on Drude model for metals, where the complex permittivity is evaluated from:

1 $${\varepsilon }_{m}(\omega )={\varepsilon }_{\infty }-\frac{{\omega }_{p}^{2}}{{\omega }^{2}+j\omega \Gamma }$$

where ω_p_ is the plasma frequency in rad/s, ${\varepsilon }_{\infty }$ is the permittivity at very high frequencies, ω is the frequency in rad/s, and Γ is the collision frequency in rad/s:

2 $$\Gamma =q/{m}^{*}\mu$$

where m* is the electron effective mass, µ is the carrier mobility and q is the electron charge. The plasma frequency is given by:

3 $${\omega }_{p}=\sqrt{{N}_{d}{q}^{2}/{\varepsilon }_{0}{m}^{*}}$$

where N_d_ is the free carrier concentration, and ε_0_ is the free-space permittivity. The plasmonic resonance of the doped Si is tuned to the mid infrared above 3 μm for a high doping concentration of 5 × 10^20^ cm^-3^, while the values of the Drude model parameters were chosen as ${\varepsilon }_{\infty }$=11.7, ω_p_ = 2.47 × 10^15^ rad/s, and Γ = 9.4 × 10^9^ rad/s^6^.

### Metal–insulator-metal bus waveguide

**Figure 1:**
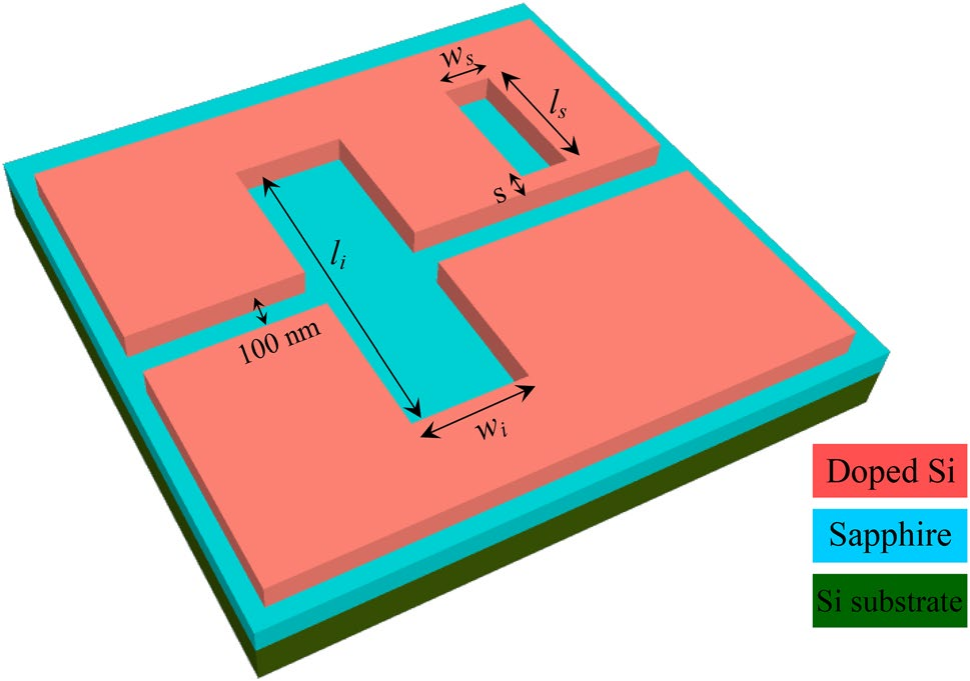
3D schematic of the Fano resonance-based sensor.

**Figure 2:**
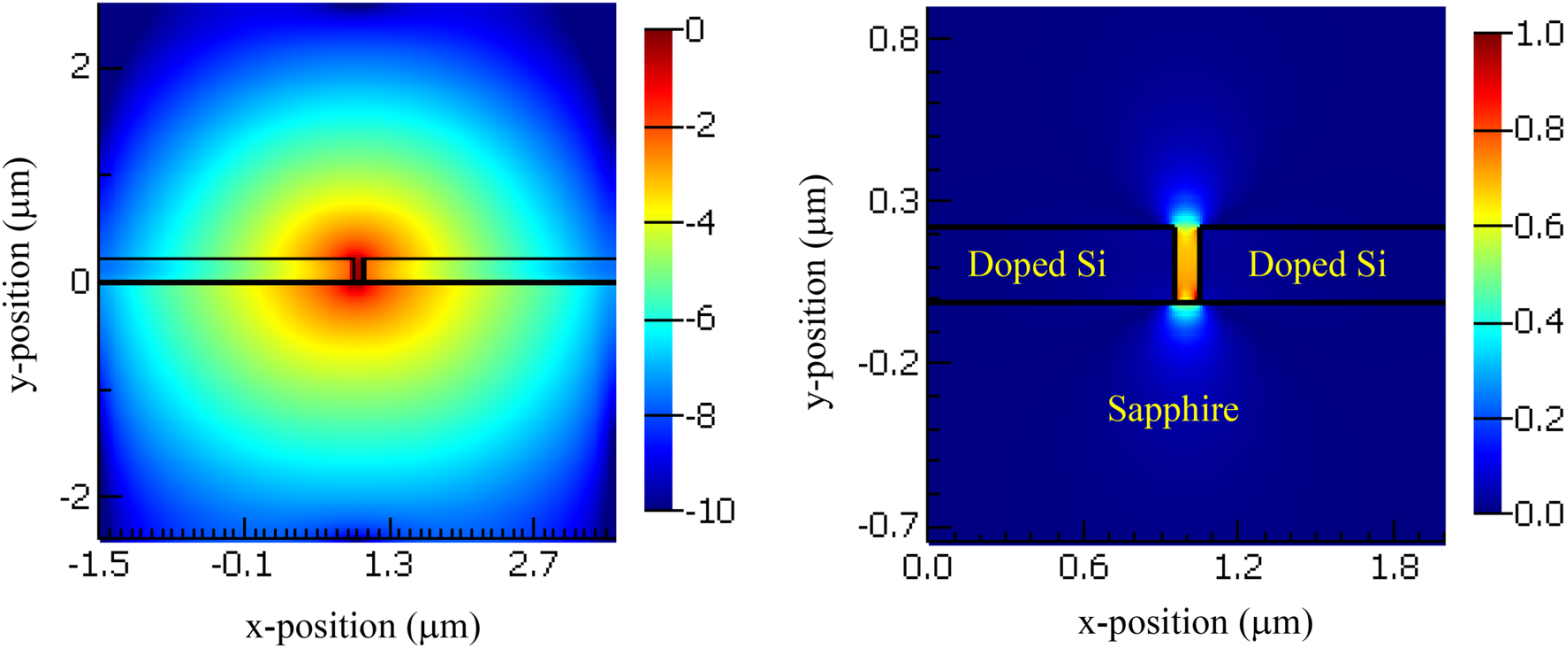
Plasmonic slot effective mode; (**a**) electric field intensity (log scale), (**b**) electric field component |Ex| (linear scale).

A doped Si wafer of thickness 220 nm on a 3 μm thick sapphire substrate is etched to form the bus waveguide of width 100 nm as shown in Fig. 1. The Si layer is doped by phosphorus with a doping concentration of 5 × 10^20^ cm^-3^ such that its plasma resonant wavelength reaches 3 μm^6^, thereby enabling plasmonic properties at wavelengths in the mid infrared spectral range. A commercial waveguide simulator based on the Finite Difference Eigenmode “FDE” solver^26^ was used to calculate the modal properties of the metal–insulator-metal bus waveguide. The FDE solver window was made large enough to enable the electric field intensity to decay to −10 on the log scale, i.e., at the simulation window boundaries ($\mathrm{log}\left({E}_{x}^{2}+{E}_{y}^{2}+{E}_{z}^{2}\right)=-10$), as shown in Fig. 2a. This was achieved for a length × width equal to 5 μm × 5 μm. At an excitation wavelength of 5 μm, metallic boundary conditions, and mesh steps of 10 nm in both x and y axes, the plasmonic slot waveguide is characterized by a complex effective index of 2.34 + j 3 × 10^–5^, and a modal loss of 3.34 dB/cm.

**Figure 3:**
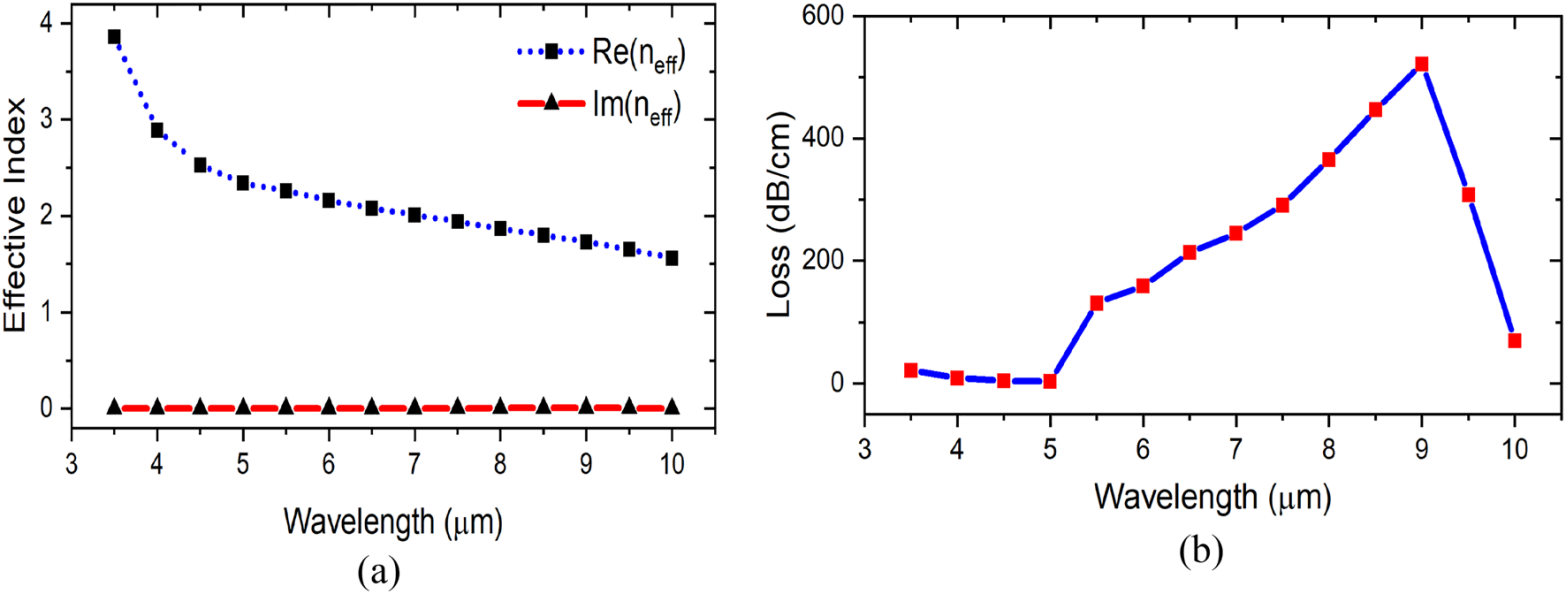
Excited plasmonic mode dispersion curve.

The electric field component |Ex| of the excited plasmonic mode is shown in Fig. 2b, the electric field shows strong is confinement in the plasmonic slot with a little power dissipation to the sapphire substrate and to the gas upwards. Moreover, we study the dispersion and the propagation loss of the excited plasmonic mode in the mid infrared range of 4–10 μm as shown in Fig. 3a and b, respectively.

### In-line rectangular cavity resonator

**Figure 4:**
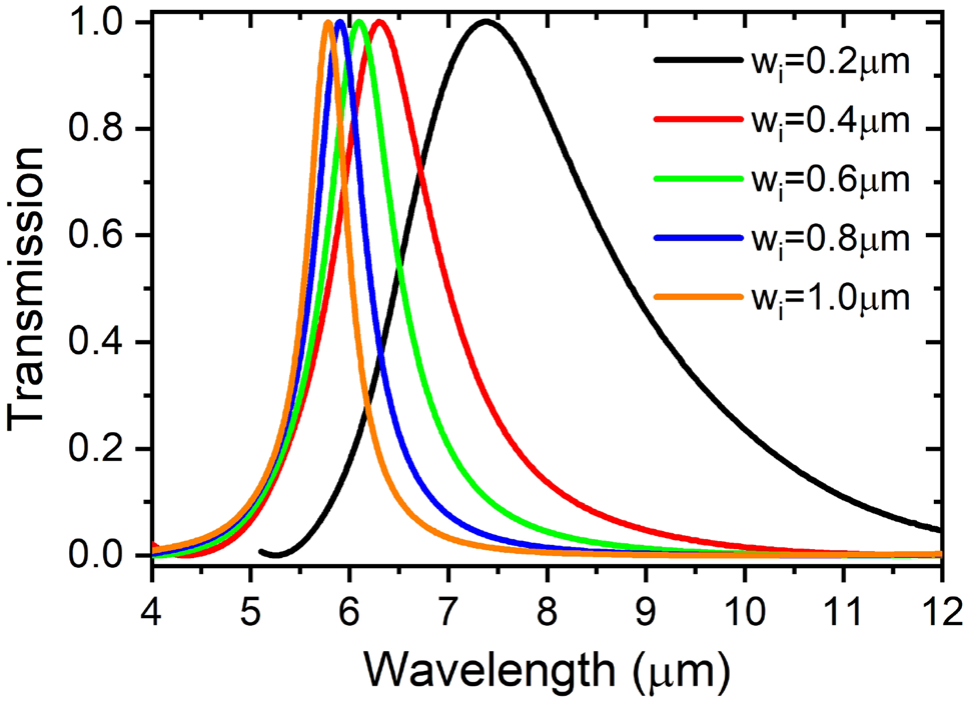
In-line rectangular cavity band bass filter response, *l*_i_ = 5 μm.

The FDTD calculations were performed using an electromagnetic simulator^27^ to calculate the response and analyze the performance of the proposed designs. A simulation time of 20,000 fs was sufficient to allow all fields including the cavity resonant fields to drop to zero by the end of the simulation process. An auto non-uniform mesh type was implemented which has the highest possible accuracy setting with a minimum mesh step of 0.25 nm. To minimize fields reflections back to the simulation region, 64 uniaxial anisotropic perfectly matched layers were used for the boundary conditions. The response of the in-line rectangular cavity resonator has the form of a bandpass filter^23^ as shown in Fig. 4, with the spectral band becomes wider for smaller rectangular widths due to the larger interaction of the fields with the metal boundaries.

**Figure 5:**
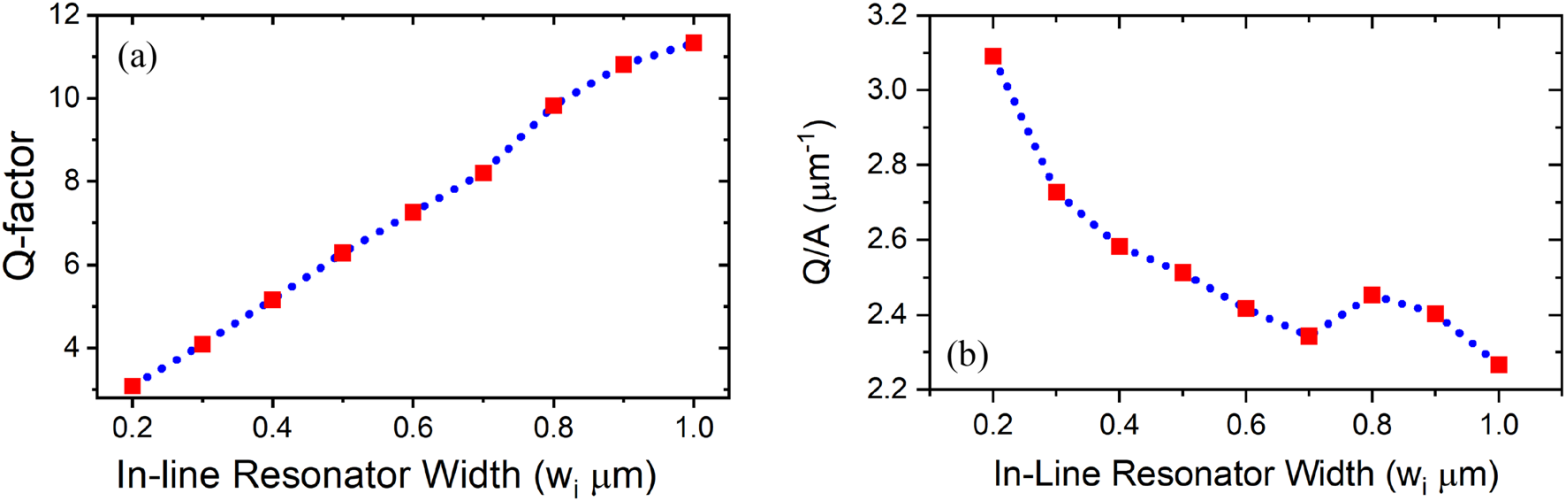
In-line resonator (**a**) Q-factor, (**b**) Q/A with in-line cavity width.

**Figure 6:**
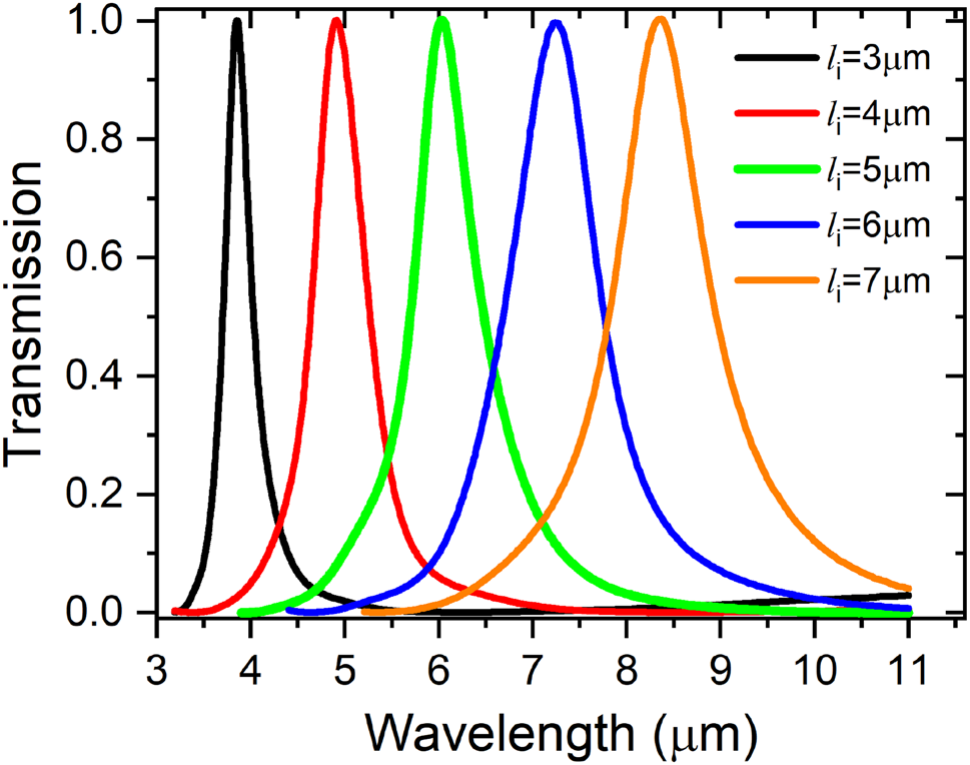
Resonance wavelength shift with in-line cavity length, w_i_ = 0.7 μm.

The Quality-factor defined by (Q = λ/Δλ) where λ is the central wavelength, and Δλ is the FWHM of the resonance band, is shown in Fig. 5, where the Q-factor shows a linear relation with the in-line rectangular resonator width. The length of the rectangular resonator controls the central position of the resonance band as shown in Fig. 6.

**Figure 7:**
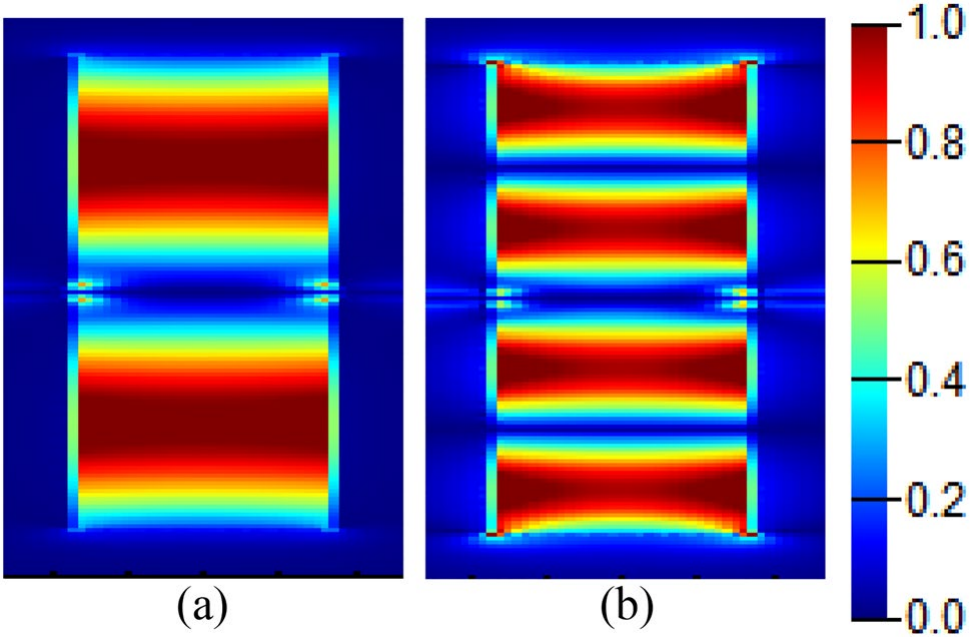
Electric field |Ex| distribution showing two different resonance orders in the in-line resonant cavity with dimensions w_i_ = 0.7 μm, *l*_*i*_ = 5 μm, at (**a**) λ = 6 μm, and (**b**) λ = 3.3 μm.

The electric field distribution within the rectangular cavity is studied at two different resonant orders as shown in Fig. 7, where strong confinement of the electric field component Ex in the rectangular cavity is observed.

### Stub rectangular cavity resonator

**Figure 8:**
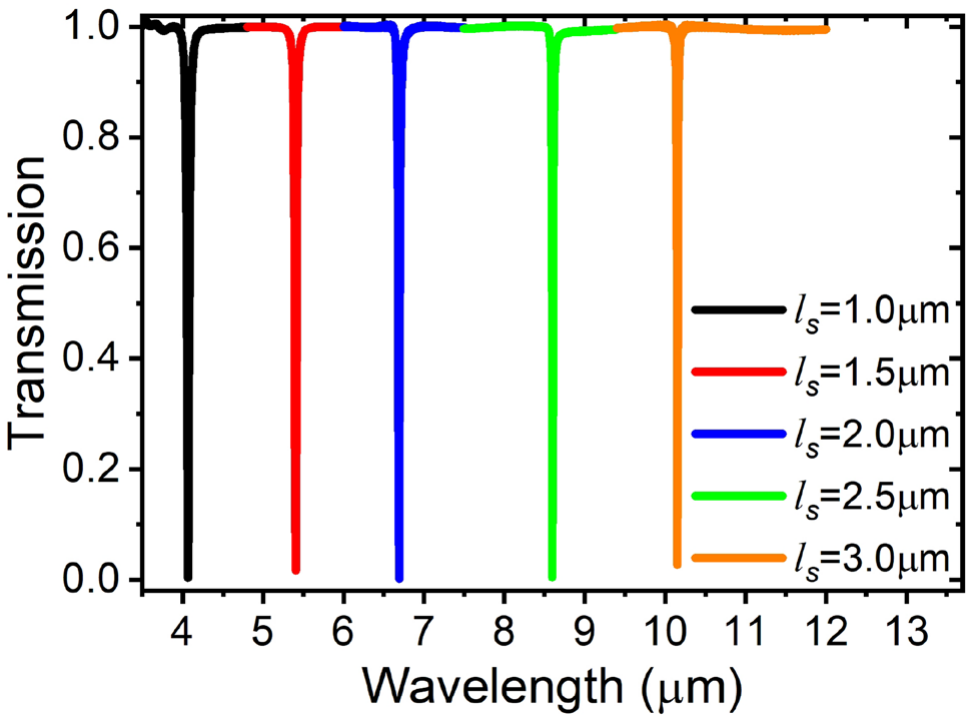
Stub rectangular cavity sharp resonances, ws=200 nm.

The stub rectangular cavity also shows distinct resonance orders but of much sharper lines as shown in Fig. 8, and higher Q-factors. The Q-factor of the 200 nm wide stub waveguide resonance line reaches 350, while that of the in-line resonator of the same width was only 3.

**Figure 9:**
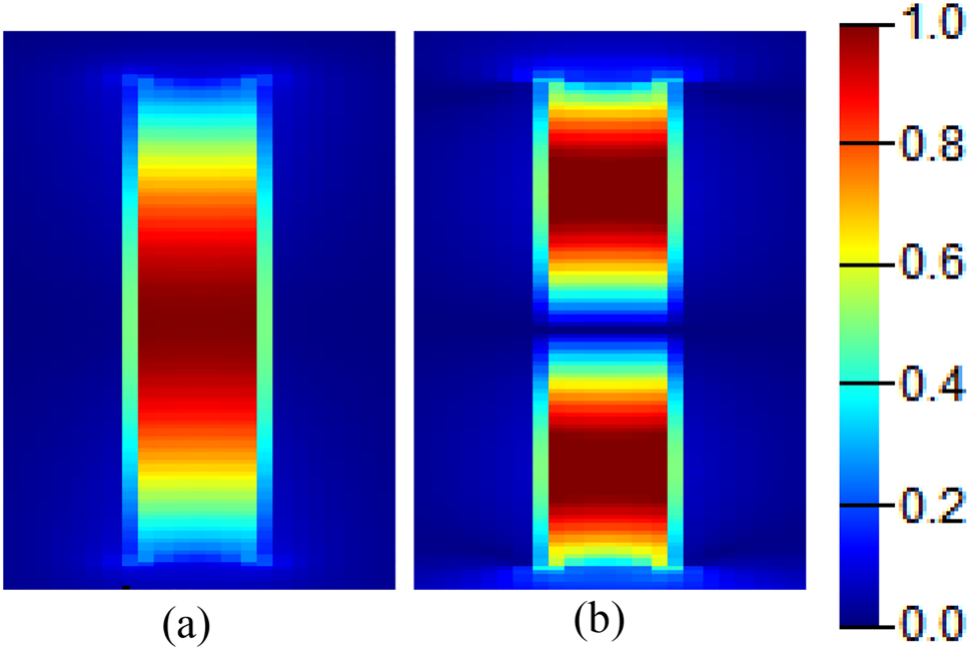
Electric field |Ex| distribution showing the first two resonant orders in the stub resonant cavity with dimensions s = 0.2 μm, w_s_ = 0.2 μm, *l*_*s*_ = 2.2 μm, at (**a**) λ = 7.1 μm, and (**b**) λ = 3.95 μm.

Figure 9 shows the electric field component Ex distribution within the stub resonator for two different resonance orders. The stub resonator is characterized by its sharp resonances, where its wavelength positions are given by:

4 $${\lambda }_{m}=\frac{2{l}_{s}{n}_{eff}}{m}$$

where *l*_s_ is the stub length, n_eff_ is the plasmonic slot mode effective index, and m is the resonance order.

## Fano resonance excitation

**Figure 10:**
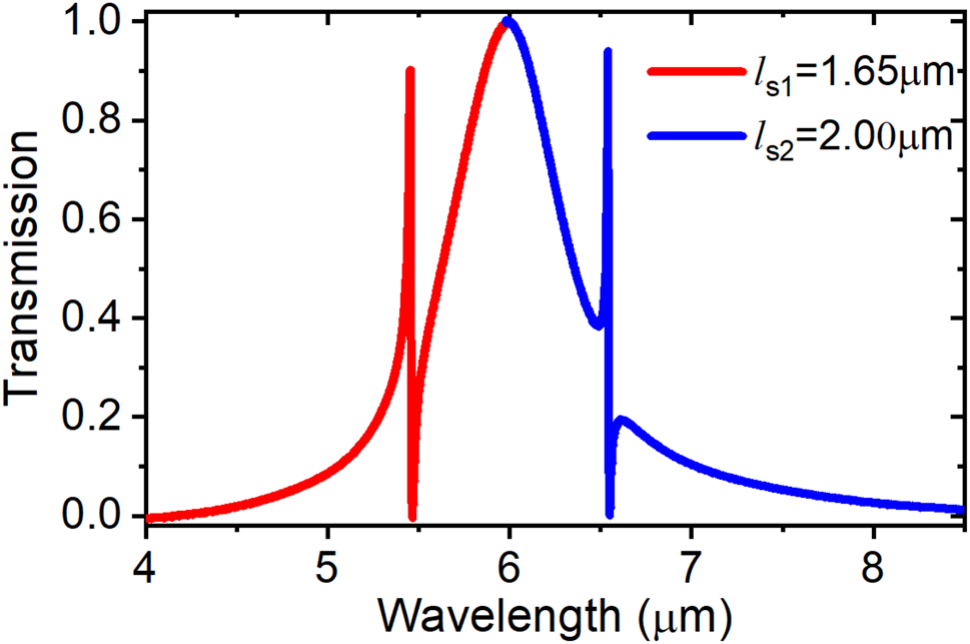
Fano resonance excited at two different spectral positions in response of coupling the in-line resonator and the stub resonator at close frequencies, w_i_ = 0.7 μm, *l*_*i*_ = 5 μm, w_s1_ = w_s2_ = 0.2 μm, and s_1_ = s_2_ = 0.2 μm.

The Fano resonance is the result of coupling a discrete localized state to a continuum of states, for example when two oscillators with strongly different damping rates with broad and narrow spectral lines are coupled together^17^. To excite the Fano resonance in our structure, we integrate the in-line and stub resonators on the same structure and optimize them “based on our studies in the previous sections” to resonate at close frequencies, such that the sharp resonance of the stub resonator couples with the decaying tail of the broader resonance of the in-line resonator as shown in Fig. 10, which shows the excitation of the Fano resonance at 5.5 μm and 6.5 μm wavelengths with an insertion loss of 0.45 dB.

**Figure 11:**
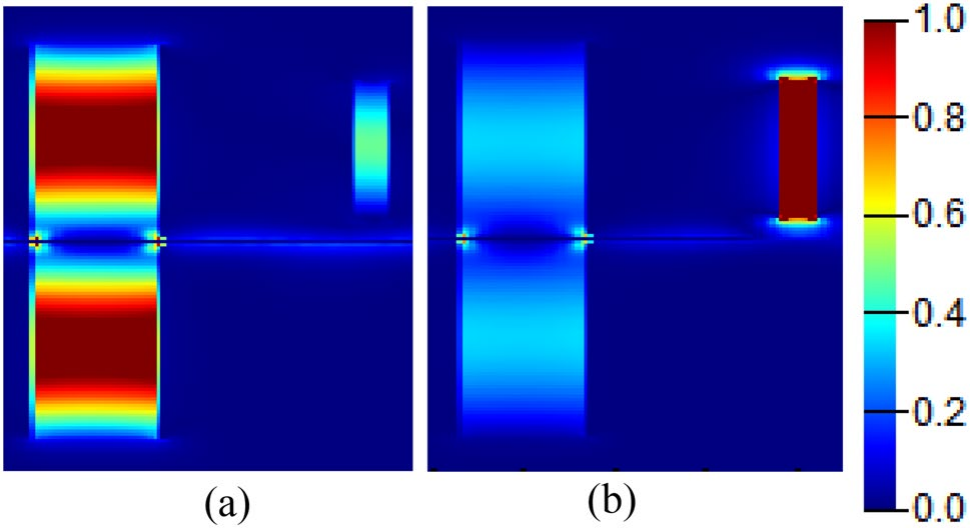
Electric field |Ex| distribution within the in-line and stub resonators at the Fano resonance wavelength at (**a**) λ = 6 μm, and (**b**) λ = 6.5 μm.

Figure 11 shows the electric field distribution in the resonators when the Fano resonance is excited, where it can be observed that at λ = 6 μm, at the top of the broader line, the field is stronger and confined in the in-line cavity. While at the Fano resonance position λ = 6.5 μm, the field is mainly confined in the stub resonator, while the in-line cavity possesses weaker field that corresponds to the decaying tail.

**Figure 12:**
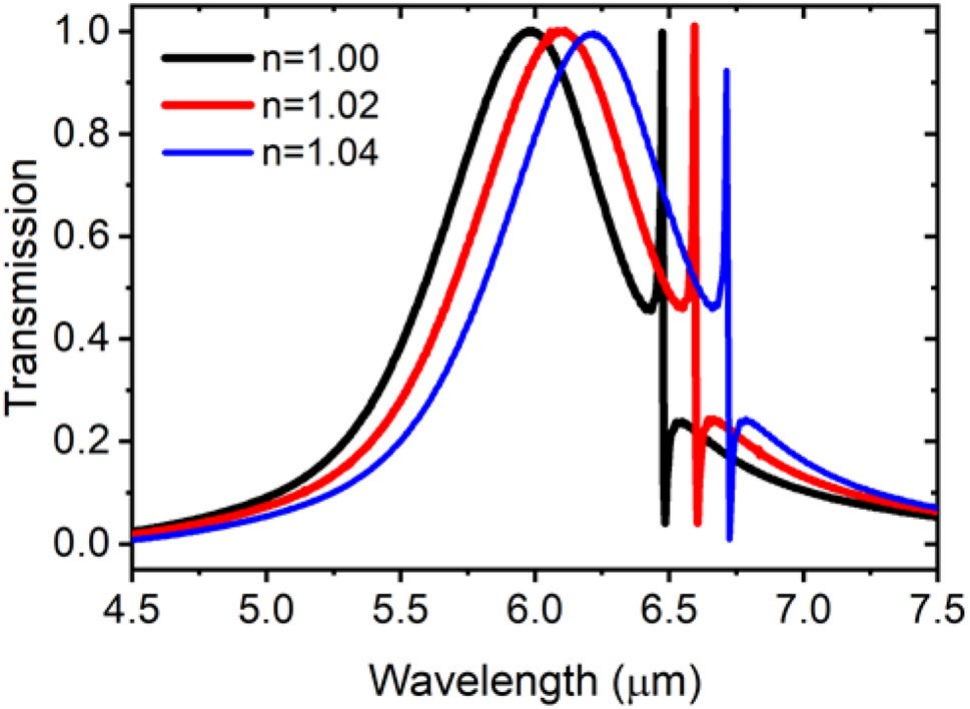
Fano resonance redshift with increasing surrounding gas index.

The integrated and coupled resonators structure can be used in sensing applications utilizing the sharpness and high sensitivity of the Fano resonance. The performance and spectral response of the sensor are studied at the 6.5 μm wavelength by varying the surrounding medium refractive index as shown in Fig. 12. The spectral sensitivity “S” defined by the resonant wavelength shift “Δλ” in response to changes in the surrounding gas refractive index “Δn” measured in refractive index unit “RIU”, i.e., (S = Δλ/Δn). Moreover, we define the Figure Of Merit “FOM” of the sensor as the spectral sensitivity divided by the Full Width at Half Maximum “FWHM” of the resonant spectral line, such that (FOM = S/FWHM). Calculations show that a sensitivity of 6000 nm/RIU can be achieved, while the FOM reaches 353, with a Q-factor of 385.

A comparison between our proposed sensor and recently published Fano sensors in the mid infrared range is demonstrated in Table 1, which shows that our sensor possesses a high sensitivity with a fairly simple design while being CMOS compatible as doped Si and not metals was used for plasmonic effects excitation.

### Simultaneous-gas sensing in the mid infrared spectral range

**Figure 13:**
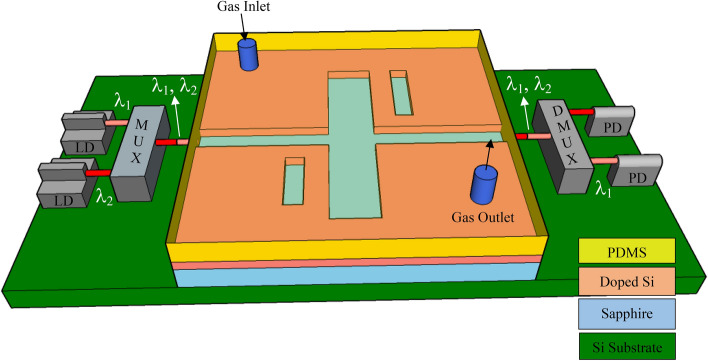
3D schematic of the Fano resonance-based sensor with two stub cavities for simultaneous detection of CH_2_O and N_2_O.

**Figure 14:**
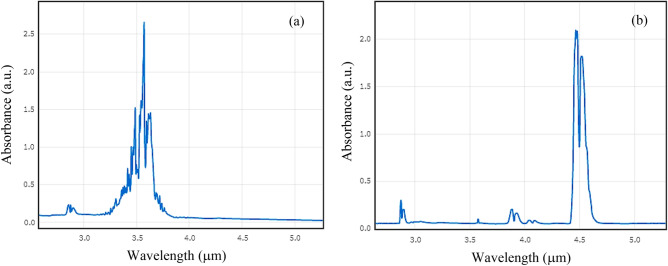
Infrared absorption spectra of (**a**) CH_2_O gas, and N_2_O gas. Adapted from^24,25^.

**Figure 15:**
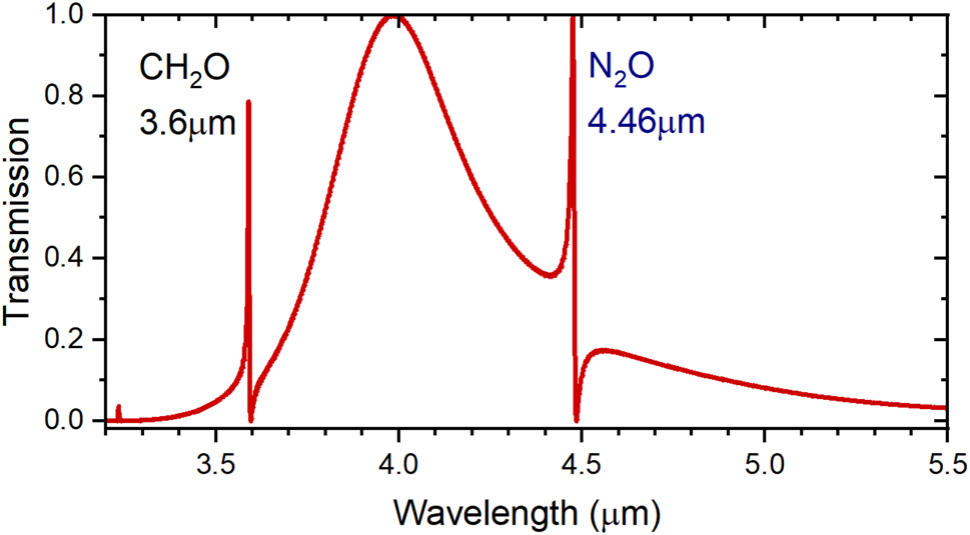
Detection of CH_2_O and N_2_O at 3.6 μm and 4.46 μm wavelengths, respectively.

Many gases have their strong absorption bands “fingerprints” in the mid infrared. So, for sensing such gases, we excite the highly sensitive Fano resonance within the absorption bands of the target gases. Simultaneous sensing of different gases is achieved by developing the sensor design to include two stub microcavities in addition to the in-line resonator. The waveguide and resonators are covered by a layer of Polydimethylsiloxane (PDMS) with a gas inlet and outlet channels. At the input of the sensor; a Multiplexer (MUX) combines different wavelength signals (λ_1_ and λ_2_) of two laser diodes (LD), similarly, a Demultiplexer (DMUX) distributes the output signals of the sensor to the photodiodes (PD) as shown in Fig. 13. As we have discussed previously, the in-line resonator provides the broad spectrum that will be perturbed at two different wavelength positions resulting in the Fano resonance. Here, we target the detection of two gases of special importance. Firstly, the colorless, flammable Formaldehyde gas CH_2_O which is found in building materials, medical preservatives, fertilizers, and pesticides^28^. Where high levels of exposure to CH_2_O gas could cause some types of cancer^29^. Secondly, the odorless, colorless Nitrous Oxide N_2_O gas supports combustion and its inhalation causes euphoria and body relaxation^30^. So, the monitoring and detection of these two gases in environments where they are produced is of great importance. CH_2_O and N_2_O are detected through their absorption bands at 3.6 μm and 4.46 μm, respectively^24,25^ shown in Fig. 14. The transmission spectrum of the multi-gas sensor is shown in Fig. 15, with optimized dimensions of the new structure as w_s1_ = 0.2 μm, *l*_s1_ = 0.85 μm, w_s2_ = 0.2 μm, *l*_s2_ = 1.3 μm, and w_i_ = 0.4 μm, *l*_i_ = 2.9 μm. The sensitivity and FOM were calculated at both wavelengths of 3.6 μm, and 4.46 μm. At the resonance wavelength of 3.6 μm, a sensitivity of 2300 nm/RIU, and a FOM of 60 were achieved. While at the resonance wavelength of 4.46 μm, the sensitivity reached 3860 nm/RIU with a calculated FOM of 145.

## Fabrication tolerance

**Figure 16:**
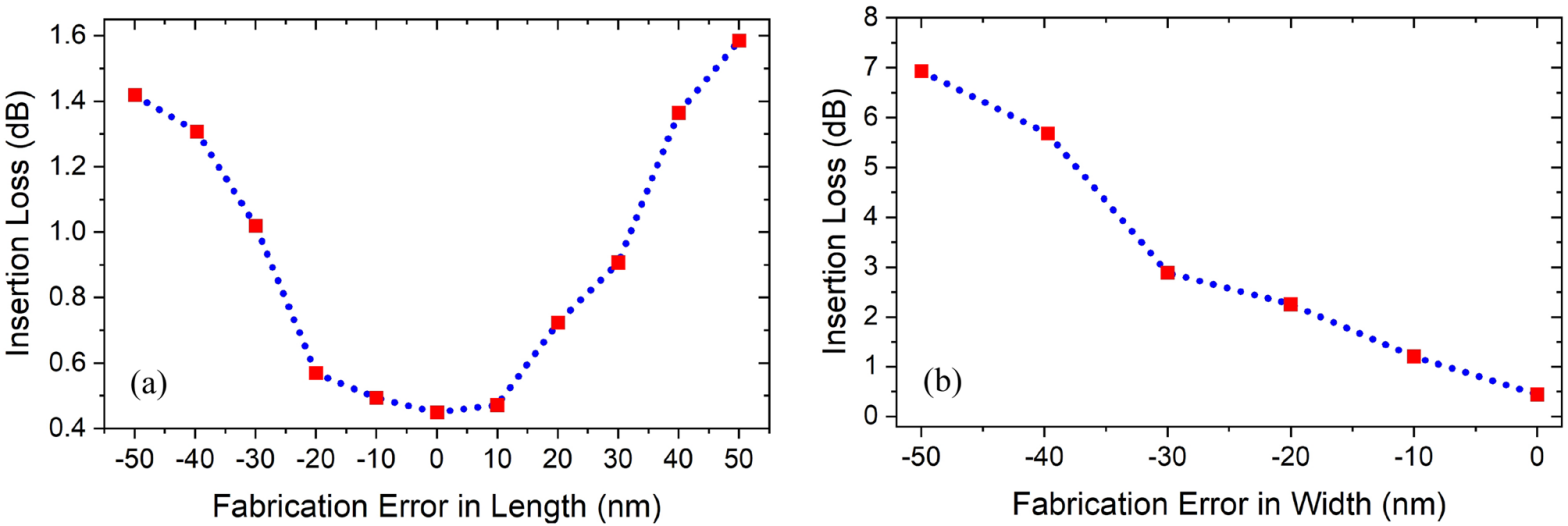
Fabrication tolerance represented by insertion loss measurements with the fabrication errors in length (**a**) and width (**b**) of the s = 0.2 μm, w_s_ = 0.2 μm, *l*_i_ = 0.8 μm stub microcavity.

**Figure 17:**
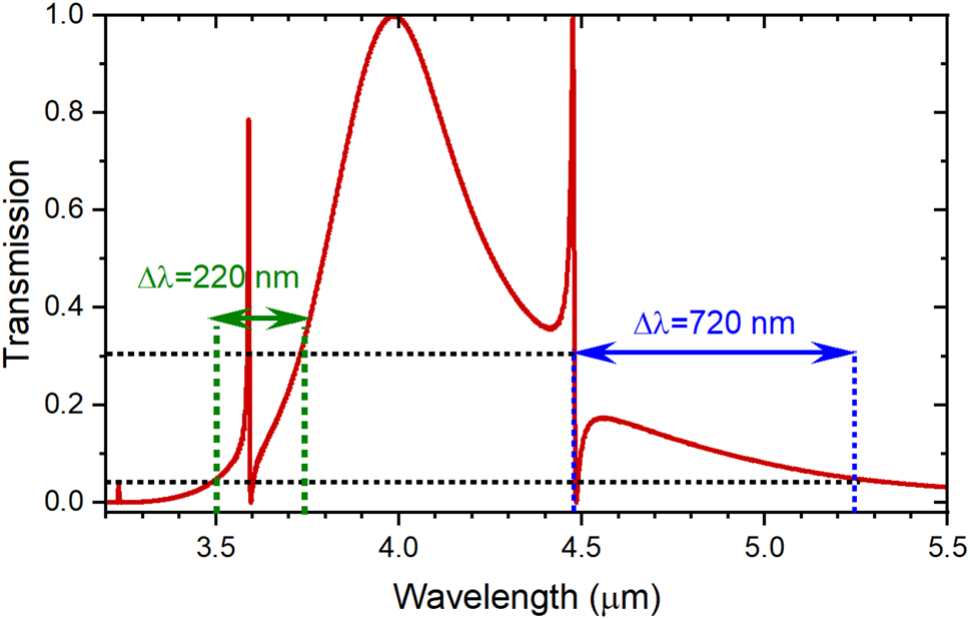
Fabrication tolerance range defined between FWGF5% and FWGF30% for both short wavelength side of spectrum (in green), and long wavelength side of spectrum (in blue), w_s1_ = 0.2 μm, *l*_s1_ = 0.85 μm, w_s2_ = 0.2 μm, *l*_s2_ = 1.3 μm, and w_i_ = 0.4 μm, *l*_i_ = 2.9 μm.

Different etching methods are typically used for etching Si wafers after photolithography or electron beam lithography^31^, such as reactive ion etching^32,33^, and inductively coupled plasma dry etching^34^. Hereby, due to its high resolution, we recommend using e-beam lithography to define the metal–insulator-metal waveguide, the rectangular resonator, and the stub resonator. The stub microcavity is obviously less fabrication tolerant than the larger rectangular cavity, so we study the fabrication tolerance of the stub microcavity of dimensions s = 0.2 μm, w_s_ = 0.2 μm, and *l*_i_ = 0.8 μm in Fig. 16, where its dimensions were changed by ± 10 nm till a fabrication tolerance of 50 nm is reached. While studying the resonators response with the changes in the stub width, it was noticed that the Fano resonance is no longer recognizable for larger widths due to the domination of the wide resonance line of the rectangular cavity, so we limit Fig. 16b to widths with −50 nm fabrication errors. Since the Fano resonance results from the coupling of the sharp stub resonance with the tail of the wider resonance of the in-line resonator, we can define the acceptable fabrication tolerance region of the stub resonator as that wavelength range of the wider resonance tail that would still results in a Fano resonance as a result of coupling with the sharper stub resonance. Qualitatively, this can be defined from the Full Width at a Given Fraction of the Maximum (FWGF). In our case, we choose the fabrication tolerance range to be between FWGF5% and FWGF30%, i.e., Full width at 5% and 30% of the maximum, respectively. This corresponds to a range of acceptable fabrication tolerance of 220 nm for the short wavelength side of the spectrum and 720 nm for the long wavelength side of the spectrum as shown in Fig. 17.

## Conclusion

A mid infrared gas sensor was demonstrated and studied. The sensor is composed of a doped Si layer that was etched to form a plasmonic slot waveguide, in-line, and stub cavity resonators. The doping level used pushes the plasmonic resonance of the Si to 3 μm, which results in exhibiting plasmonic properties in the mid infrared range. The performance of each resonator type was investigated individually and then they were both integrated together. When coupled at close frequencies, Fano resonance was excited because of the interference between the wide response of the in-line microcavity and the sharper resonance of the stub resonator. Performance parameters were measured such as the sensitivity, FOM and insertion loss of the device.

## Data Availability

The datasets used and/or analysed during the current study available from the corresponding author on reasonable request.

## References

[1] Tittel, F. K., Richter, D., & Fried, A. Mid-infrared laser applications in spectroscopy in solid-state mid-infrared laser sources (eds. Sorokina, I. T., Vodopyanov, K. L.) Topics in Applied Physics, 89, 458– 529 (Springer, 2003).

[2] Law S, Yu L, Rosenberg A, Wasserman D (2013). All-semiconductor plasmonic nanoantennas for infrared sensing. Nano lett..

[3] Ahmadivand A, Gerislioglu B (2022). Photonic and plasmonic metasensors. Laser Photonics Rev..

[4] Maier SA, Atwater HA (2005). Plasmonics: Localization and guiding of electromagnetic energy in metal/dielectric structures. J. Appl. Phys..

[5] Gramotnev DK, Bozhevolnyi SI (2010). Plasmonics beyond the diffraction limit. Nat. Photon..

[6] Sherif SM, Swillam MA (2016). Metal-less silicon plasmonic mid-infrared gas sensor. J. Nanophotonics.

[7] Gamal R, Ismail Y, Swillam MA (2015). Silicon waveguides at the mid-infrared. J. Light. Technol..

[8] Soref R, Peale RE, Buchwald W (2008). Longwave plasmonics on doped silicon and silicides. Opt. Express.

[9] Sherif SM, Elsayed MY, Shahada LA, Swillam MA (2019). Vertical silicon nanowire-based racetrack resonator optical sensor. Appl. Phys. A.

[10] El Shamy RS, Khalil D, Swillam MA (2020). Mid infrared optical gas sensor using plasmonic Mach-Zehnder interferometer. Sci. Rep..

[11] Ahmadivand A, Gerislioglu B, Ahuja R, Mishra YK (2020). Terahertz plasmonics: The rise of toroidal metadevices towards immunobiosensings. Mater. Today.

[12] Kravets VG, Kabashin AV, Barnes WL, Grigorenko AN (2018). Plasmonic surface lattice resonances: A review of properties and applications. Chem. Rev..

[13] Tan TC, Srivastava YK, Ako RT, Wang W, Bhaskaran M, Sriram S, Al-Naib I, Plum E, Singh R (2021). Active Control of nanodielectric-induced THz Quasi-BIC in flexible metasurfaces: A platform for modulation and sensing. Adv. Mater..

[14] Rahmani M, Luk'yanchuk B, Hong M (2013). Fano resonance in novel plasmonic nanostructures. Laser Photonics Rev..

[15] Miroshnichenko AE, Flach S, Kivshar YS (2010). Fano resonances in nanoscale structures. Rev. Mod. Phys..

[16] Sherif SM, Zografopoulos DC, Shahada LA, Beccherelli R, Swillam M (2017). Integrated plasmonic refractometric sensor using Fano resonance. J. Phys. D Appl. Phys..

[17] Limonov MF, Rybin MV, Poddubny AN, Kivshar YS (2017). Fano resonances in photonics. Nat. Photon..

[18] Hong Q, Luo J, Wen C, Zhang J, Zhu Z, Qin S, Yuan X (2019). Hybrid metal-graphene plasmonic sensor for multi-spectral sensing in both near-and mid-infrared ranges. Opt. Express.

[19] Zhang J, Hong Q, Zou J, He Y, Yuan X, Zhu Z, Qin S (2020). Fano-resonance in hybrid metal-graphene metamaterial and its application as mid-infrared plasmonic sensor. Micromachines.

[20] Ahmed AM, Mehaney A (2019). Ultra-high sensitive 1D porous silicon photonic crystal sensor based on the coupling of Tamm/Fano resonances in the mid-infrared region. Sci. Rep..

[21] Chau YFC (2020). Mid-infrared sensing properties of a plasmonic metal–insulator–metal waveguide with a single stub including defects. J. Phys. D Appl. Phys..

[22] Zhang Y, Liang Z, Meng D, Qin Z, Fan Y, Shi X, Smith DR, Hou E (2021). All-dielectric refractive index sensor based on Fano resonance with high sensitivity in the mid-infrared region. Results Phys..

[23] Kotb R, Ismail Y, Swillam MA (2013). Integrated metal-insulator-metal plasmonic nano resonator: an analytical approach. Prog. Electromagn. Res. Lett..

[24] National Institute of Standards and Technology website, Formaldehyde gas absorption spectrum, https://webbook.nist.gov/cgi/cbook.cgi?ID=C50000&Type=IR-SPEC

[25] National Institute of Standards and Technology website, Nitrous gas absorption spectrum, https://webbook.nist.gov/cgi/cbook.cgi?ID=C10024972&Type=IR-SPEC

[26] Lumerical Inc. https://www.lumerical.com/products/mode/

[27] Lumerical Inc. https://www.lumerical.com/products/FDTD/

[28] https://www.epa.gov/formaldehyde/facts-about-formaldehyde#whatare

[29] https://www.cancer.gov/about-cancer/causes-prevention/risk/substances/formaldehyde

[30] https://www.ncbi.nlm.nih.gov/books/NBK532922/

[31] Kato K, Liu Y, Murakami S, Morita Y, Mori T (2021). Electron beam lithography with negative tone resist for highly integrated silicon quantum bits. Nanotechnology.

[32] Doll PW, Al-Ahmad A, Bacher A, Muslija A, Thelen R, Hahn L, Ahrens R, Spindler B, Guber AE (2019). Fabrication of silicon nanopillar arrays by electron beam lithography and reactive ion etching for advanced bacterial adhesion studies. Mater. Res. Express..

[33] Huff M (2021). Recent advances in reactive ion etching and applications of high-aspect-ratio microfabrication. Micromachines.

[34] Goodyear AL, Mackenzie S, Olynick DL, Anderson EH (2000). High resolution inductively coupled plasma etching of 30 nm lines and spaces in tungsten and silicon. J. Vac. Sci. Technol. B Microelectron. Nanometer. Struct. Process. Meas. Phenom..

